# Food insecurity, COVID-19 and diets in Fiji – a cross-sectional survey of over 500 adults

**DOI:** 10.1186/s12992-023-01004-w

**Published:** 2023-12-11

**Authors:** Jacqui Webster, Anasaini Moala, Briar McKenzie, Joseph Alvin Santos, Aliyah Palu, Alvina Deo, Susana Lolohea, Mohammed Sanif, Penaia Naivunivuni, Shajal Kumar, Emosi Vimatemate, Helen Tawakilai, Litiana Seru, Mark Woodward, Dori Patay, Devina Nand, Ateca Kama, Erica Reeve, Gade Waqa, Colin Bell

**Affiliations:** 1 The George Institute for Global Health, UNSW, Sydney, Australia; 2 C-POND, Fiji National University, Suva, Fiji; 3 Ministry of Health, Suva, Fiji; 4 The George Institute for Global Health, Imperial College, London, UK; 5 Menzies Centre for Health Policy and Economics, Charles Perkins Centre, The University of Sydney, Sydney, Australia; 6 Institute for Health Transformation, Deakin University, Geelong, Australia

**Keywords:** Food security, Diet, Nutrition, Pacific islands, Remote and regional communities, Covid-19, Small island developing nations (SIDS)

## Abstract

**Introduction:**

Food insecurity is associated with inadequate nutrition and increased rates of chronic disease. The primary aim of this study was to assess self-reported food insecurity and the perceived impact of COVID-19 on food security, in two regional districts of Central Fiji, as part of a broader program of work on strengthening and monitoring food policy interventions. The secondary aim was to explore the relationship between food insecurity and salt, sugar and fruit and vegetable intake.

**Methods:**

Seven hundred adults were randomly sampled from the Deuba and Waidamudamu districts of Viti Levu, Fiji. Interview administered surveys were conducted by trained research assistants with data collected electronically. Information was collected on demographics and health status, food security, the perceived impact of COVID-19 on food security, and dietary intake. Food insecurity was assessed using nine questions adapted from Fiji’s 2014/5 national nutrition survey, measuring markers of food insecurity over the last 12 months. Additional questions were added to assess the perceived effect of COVID-19 on responses. To address the secondary aim, interview administered 24-hour diet recalls were conducted using Intake24 (a computerised dietary recall system) allowing the calculation of salt, sugar and fruit and vegetable intakes for each person. Weighted linear regression models were used to determine the relationship between food insecurity and salt, sugar and fruit and vegetable intake.

**Results:**

534 people participated in the survey (response rate 76%, 50.4% female, mean age 42 years). 75% (75.3%, 95% CI, 71.4 to 78.8%) of people reported experiencing food insecurity in the 12 months prior to the survey. Around one fifth of people reported running out of foods (16.8%, 13.9 to 20.2%), having to skip meals (19.3%, 16.2 to 22.9%), limiting variety of foods (19.0%, 15.9 to 22.5%), or feeling stressed due to lack of ability to meet food needs (19.5%, 16.4 to 23.0%). 67% (66.9%, 62.9 to 70.7%) reported becoming more food insecure and changing what they ate due to COVID-19. However, people also reported positive changes such as making a home garden (67.8%, 63.7 to 71.6%), growing fruit and vegetables (59.5%, 55.6 to 63.8%), or trying to eat healthier (14.7%, 12.0 to 18.0%). There were no significant associations between food insecurity and intakes of salt, sugar or fruit and vegetables.

**Conclusion:**

Participants reported high levels of food insecurity, exceeding recommendations for salt and sugar intake and not meeting fruit and vegetable recommendations, and becoming more food insecure due to COVID-19. Most participants reported making home gardens and/or growing fruit and vegetables in response to the pandemic. There is an opportunity for these activities to be fostered in addressing food insecurity in Fiji, with likely relevance to the Pacific region and other Small Island Developing States who face similar food insecurity challenges.

**Supplementary Information:**

The online version contains supplementary material available at 10.1186/s12992-023-01004-w.

## Introduction

Health security in Pacific Countries and Territories is closely related to food security – the availability of and access to healthy and affordable food. Food security at a national level is achieved through local food production and food importation. Local production is increasingly threatened by climate change, young people’s diminishing interest in agriculture, and the aging population [1, 2]. Food imports are influenced by price and availability fluctuations, as occurred during the global financial crisis in 2008 and more recently due to constraints related to the COVID-19 pandemic [3]. Trade agreements and efforts to support subsistence and commercial food production, for example through fishing [4], are in place across the Pacific but they are hampered by a lack of regional coordination and development assistance, and they seldom affect the livelihoods of the most vulnerable such as those living on outer Islands or in peri-urban areas [5]. This means that most Pacific countries are vulnerable to food security threats.

Food insecurity has been adversely associated with non-communicable diseases (NCDs) risk [6] and obesity [7] in low- and middle-income countries (LMICs), including some Pacific Island Countries. There is a high prevalence of NCDs and related risk factors in Fiji and the Pacific Island region more broadly. NCDs account for over 75% of mortality in Pacific Island Countries, with high rates of premature mortality from NCDs and high proportions of people living with debilitating chronic diseases [8]. From 2011 data, 42.0% of women and 22.4% of men live with obesity, and a third of adults have high blood pressure in Fiji [9]. The link between food insecurity and obesity in LMICs is thought to be mediated by the comparatively low cost of packaged, high-energy foods, although other dietary risk factors, for example, high intakes of salt, saturated and trans-fat and low intakes of fruit, vegetables and fibre, are also associated with food insecurity [10]. A study from Samoa reported high rates of consumption of sugary and fatty energy dense foods along with high rates of food insecurity [11], with cost reported as a major driver of food choice. Similarly, a study from the Solomon Islands reported high rates of food insecurity among peri-urban women and dietary patterns high in processed and sugary foods, especially for those with limited land access and low incomes [12].

Food insecurity compromises Fiji’s development agenda [13]. Consequently, the Government has committed to food systems transformation through its participation in the UN Food Systems Summit [14], and is working to develop new multisectoral plans for NCD prevention and nutrition and food security. Developing an understanding of contributors to all forms of malnutrition in Fiji provides an evidence base that can inform the policy process. Current food security data is vital for this endeavor; however, the last representative survey of food security in Fiji was conducted in 2014/15 [15]. Furthermore, a better understanding of how dietary intakes are shaped by food security is needed to inform evidenced-based food policy in Fiji.

The primary aim of this paper was to describe self-reported food security in Fijian adults aged 18 years or older, including how consumption patterns may have changed in response to COVID-19. A secondary aim was to examine associations between food security and dietary intakes.

## Methods

This was a cross-sectional study within the central division of Fiji [16]. Questions on food security and perceived effect of COVID-19 on food security were within a broader survey on dietary intake and knowledge, attitudes and behaviours [16, 17]. The project received ethics clearance from the University of New South Wales (# HC200469) and Fiji National University College of Health Research Ethical Committee (CHREC264.20). This study follows the STROBE reporting guidelines for cross-sectional studies [18], Supplementary Table 1.

### Sample size and recruitment

The Central Division of Fiji is home to approximately 42% of the population of Fiji, and is part of the main island of Viti Levu, where 80% of the population live. For this survey, two enumeration areas were randomly selected. There were 836 enumeration areas categorised as either “rural” or “urban” by the Fiji Bureau of Statistics. To blind the selection, these enumeration areas were coded, one urban and one rural enumeration area was then selected. The chosen areas were uncoded following selection. The sample size of 600 (300 in each area) was chosen to achieve at least 80% power, using 5% significance tests, to detect a 0.6 g/day change in salt intake (SD 3.6) and a 0.9 absolute percent change in free sugar intake (SD 5.4) when the survey is repeated. This sample size was calculated based on a test for two means [16, 19], adjusted for clustering and stratification (DE of 1.5). Assuming an estimated 16% non-response rate [16], 700 people were sampled, with one adult (aged 18 years or older) per household randomly selected. The census data available to the research team during the survey preparation was from 2012 and considered out of date given 10 years had passed at the time of the present survey. As such, comprehensive household listing was undertaken to support the sample selection. Prior to beginning recruitment, approvals were sought from the Provincial Administrators and the local village chiefs were contacted to request permission to enter the village and conduct the research. Researchers then visited the houses of selected participants to inform them of the study and invite them to participate. If no-one was home, or only a person under 18 was home, then a repeat visit was made at a later stage. Eligibility was based on age (aged 18 years or older), and the ability to provide informed consent. Participant information sheets and consent forms were available in English, Hindi and Fijian. Each consented participant was assigned a unique ID number.

Eight research assistants from Fiji National University were trained in survey data collection procedures and interviewed the participants. The entire enrolment and data collection processes were conducted face-to-face in English (an official language spoken by more than 90% of the population). The research assistants were able to assist participants by orally translating written material into the local language when needed. The data collection process took approximately one hour and took place in the participant’s home, or an accessible communal place (for example, village hall), based on the preference of the participant. The surveys took place from March to June 2022.

### Survey measures

Surveys were interview administered, and data collected via electronic forms on tablets. A word document version of the forms was developed by the research team, containing the measures described below. This form was converted to an electronic form accessible on the tablets used by the research assistants, via a Android Package Kit file. There was one form per participant, with data entered linking to a deidentified master datasheet. The electronic form is not publicly accessible; however, it included the questions as described in this section. The survey collected information on general characteristics, such as sex, age, ethnicity, education level attained and employment status, smoking status, and alcohol consumption frequency. Questions were also asked on history of disease. Physical measures were taken for height, weight, and blood pressure by the research assistants, with measures entered in the electronic form. Further information on collection of these data is available by Silatolu et al. [17].

#### Food insecurity and perceived influence of COVID19

Food insecurity was assessed based on responses to nine questions with a tenth indicator recorded asking whether they had experienced any of the measures of food insecurity. The questions were adapted from Fiji’s previous National Food and Nutrition Survey [15], asked in the following ways: “In the last 12 months, have you experienced any of the following; Not enough food for balanced meals?; Run out of basic food (staple foods)?; Have had to have smaller or skip meals due to lack of money?; Had a limited variety of food due to lack of money?; Feel stressed because of not enough food/money for food?; Use of special food vouchers when they have not had enough money?; Feel stressed because can’t provide food to meet demands for social/cultural/religious occasions?; Feel stressed because can’t afford balanced meals for children? Questions were added on the extent to which people had changed their diets due to COVID-19 with three responses: ‘Greatly’ (became more food insecure due to COVID-19); ‘Somewhat’ (became slightly more food insecure due to COVID-19) and ‘Not at all’ (did not experience a change in food security). A range of yes/no options to provide further information on how people had changed their diets were then asked in the following ways: “Did you change what/how you ate or how you accessed food during the Covid-19 pandemic?: Stocked up on food more than you normally would have; made a home garden; grew more fruits and vegetables to eat; changed what you ate – tried to eat more “health” foods; ate less than you normally would have for health-related reasons; ate less than you normally would have due to Covid-related food shortages; ate more than you normally would have”. Responses to the questions were recorded in the electronic forms, following the questions on participant demographic characteristics and the entering of the physical measurements.

#### Dietary intake

Dietary intake was measured by 24-hour diet recall. The validated Intake24 diet recall application [17, 20–22] was used. This was adapted for Fiji from the New Zealand food composition database, by including nutrient information from the top 100 most consumed foods in Fiji, which came from the National Nutrition Survey and discussions with key stakeholders [17]. Research assistants guided participants through the three pass 24-hr diet recall to collect data on all foods and drinks consumed within the past 24 h. Images were provided for each food item in the application, to aid accuracy in reporting the dietary data entered into the application which was then automatically converted into nutrient intake data (including information on salt and sugar intake) by linking to the Intake24 food composition database. This application is accessible, by contacting Intake24 [23].

### Data analyses

Complete case analyses were conducted, after 45 people (7%) were removed from the analysis due to missing, or implausible data, based on World Health Organization NCD STEPs survey guidance [24].

Analyses were weighted to reflect the probability of individual selection (sample weight) and to match the population structure of Deuba and Waidamudamu (population weight), weighting was based on age, sex and ethnicity. The differences between subgroups, defined by sex (female vs. male), age (18 to 44 years vs. 45 years and above), ethnicity (indigenous iTaukei Fijians and Fijians of Indian descent or other), and area (Deuba vs. Waidamudamu), were compared using weighted chi-square tests. The relationship between food insecurity and salt and sugar intake, and fruit and vegetable intake, was determined using weighted linear regression, both unadjusted and adjusted for sex, age, enumeration area, ethnicity, education level, BMI classification and hypertension status. A variable for “food insecurity” for the regression analysis was generated using the nine questions. The variable was coded 1 if the participant experienced any of the 9 food insecurity questions, otherwise 0. All analyses were conducted in STATA BE V17.0, the svy command in Stata was used accounting for strata effects [25], and the Taylor linearization method was employed for variance estimation [26]. All results were reported as percentages with 95% confidence intervals (CIs).

## Results

### Demographics and health status

Seven hundred people were approached. Reasons for non-response or refusal included: participant had permanently moved (*n* = 50), refused to participate (no specific reason given, *n* = 46), too busy to participate (*n* = 15), family objected to participation (*n* = 4), deceased (*n* = 3) or unwell (*n* = 3). Forty-five people were then excluded from analyses due to implausible and missing data. Our final study population was 534 people (50% women), a response rate of 76%. Mean age was 42 years, and 63.6% (95% CI 59.4 to 67.6) were aged 18 to 44 years. Just less than half of the respondents were iTaukei (Indigenous Fijian). A third had a tertiary education or higher, and most people shared their household (i.e., lived with family and/or extended family). 28% had a history of high blood pressure and just less than 10% had a history of diabetes. About 50% (50.8, 95% CI – 46.8 to 54.8) had hypertension. Mean waist circumference was 96.5 cm (95% CI 95.0 to 98.0) Mean body mass index was 28.8 kg/m^2^ (28.2 to 29.3). Mean BMI was significantly higher for women than men (30.3 kg/m^2^ (29.4 to 31.2) vs. 27.2 kg/m^2^ (26.5 to 27.9)). Almost 70% of people were living with overweight or obesity (overweight, 27.8%,24.3 to 31.7%, obesity 41.4%,37.5 to 45.4%), and more women than men were living with obesity (Table 1).

**Table 1:** Demographic and health characteristics of nutrition survey participants (*n* = 534)

Variables	Weighted estimates	Women (*n* = 272)	Men (*n* = 262)
**Sex** (%, 95% CI)			
Female	50.36 (45.92 to 54.81)		
Male	49.64 (45.19 to 54.08)		
**Age, years** (mean, 95% CI)	41.68 (40.98 to 42.38)	42.52 (41.51–43.53)	40.83 (39.85–41.81)
**Age group** (%, 95% CI)^1^			
18 to 44 years	63.60 (59.41 to 67.60)	61.40 (55.37 to 67.09)	65.84 (59.96 to 71.27)
45 years and above	36.40 (32.40 to 40.59)	38.60 (32.91 to 44.63)	34.16 (28.73 to 40.04)
**Ethnic background** (%, 95% CI)			
iTaukei	46.32 (41.91 to 50.79)	48.60 (42.45 to 54.80)	44.01 (37.79 to 50.43)
Fijian of Indian descent or other^2^	53.68 (49.21 to 58.09)	51.40 (45.20 to 57.55)	55.99 (49.57 to 62.21)
**Area** (%, 95% CI)			
Deuba	59.96 (55.69 to 64.09)	58.45 (52.37 to 64.29)	61.50 (55.47 to 67.18)
Waidamudamu	40.04 (35.91 to 44.31)	41.55 (35.71 to 47.63)	38.50 (32.82 to 44.53)
**Education** (%, 95% CI)			
Secondary education or below	69.39 (65.41 to 73.09)	71.68 (66.13–76.64)	67.06 (61.23–72.42)
Tertiary education (University)	29.43 (25.76 to 33.38)	27.28 (22.38–32.80)	31.60 (26.32–37.40)
Postgraduate or higher	1.19 (0.53 to 2.64)	1.04 (0.35–3.05)	1.34 (0.42–4.20)
**Household type** (%, 95% CI)			
Live alone	4.17 (2.88 to 6.00)	3.85 (2.19–6.69)	4.50 (2.76–7.25)
Shared household	95.83 (94.00 to 97.12)	96.15 (93.31–97.81)	95.50 (92.75–97.24)
**Self-assessed health** (%, 95% CI)			
Excellent	21.05 (17.79 to 24.72)	19.65 (15.36–24.78)	22.47 (17.79–27.96)
Very good	33.08 (29.30 to 37.10)	32.30 (27.18–37.88)	33.88 (28.44–39.77)
Good	35.69 (31.81 to 39.76)	33.40 (28.23–39.00)	38.01 (32.35–44.02)
Fair	8.57 (6.56 to 11.13)	12.06 (8.87–16.19)	5.03 (2.92–8.53)
Poor	1.61 (0.88 to 2.92)	2.59 (1.31–5.05)	0.61 (0.17–2.25)
**Current smoker** (%, 95% CI)	28.72 (25.36 to 32.32)	14.77 (11.21–19.21)	42.87 (37.25–48.68)
Ever smoked regularly (%, 95% CI)	26.24 (22.94 to 29.83)	12.52 (9.15–16.89)	40.16 (34.57–46.02)
**Time since last alcoholic drink (%, 95% CI)**			
1 week or less	14.94 (12.18 to 18.20)	6.23 (3.85–9.93)	23.78 (18.93–29.43)
More than 1 week to less than 12 months	22.12 (18.83 to 25.81)	17.39 (13.42–22.23)	26.92 (21.84–32.69)
12 months or more	23.92 (20.49 to 27.72)	20.93 (16.59–26.05)	26.95 (21.83–32.77)
Never	39.02 (35.48 to 42.68)	55.45 (50.04–60.74)	22.35 (17.90–27.53)
**History of disease (%, 95% CI)**			
High blood pressure	27.96 (24.48 to 31.73)	28.31 (23.68–33.44)	27.61 (22.56–33.30)
Low blood pressure	9.85 (7.72 to 12.48)	15.80 (12.07–20.42)	3.82 (2.13–6.76)
High cholesterol or fat in blood	8.63 (6.59 to 11.21)	8.96 (6.12–12.93)	8.29 (5.66–11.99)
Heart attack	1.57 (0.86 to 2.85)	1.58 (0.66–3.72)	1.56 (0.68–3.53)
Stroke	0.98 (0.45 to 2.12)	0.72 (0.17–2.91)	1.24 (0.49–3.09)
Angina	12.62 (10.07 to 15.71)	12.78 (9.48–17.00)	12.46 (8.83–17.31)
Diabetes	9.72 (7.71 to 12.17)	10.33 (7.51–14.04)	9.10 (6.49–12.61)
**Anthropometric measurements**			
Systolic blood pressure, mmHg (mean, 95% CI)	136.37 (134.89 to 137.84)	136.20 (133.90 -138.50)	136.53 (134.69–138.36)
Diastolic blood pressure, mmHg (mean, 95% CI)	84.74 (83.73 to 85.75)	84.30 (82.82–85.78)	85.19 (83.82–86.56)
Hypertension^3^ (%, 95% CI)	49.21 (44.77 to 53.66)	To add	To add
Height, cm (mean, 95% CI)	165.78 (165.08 to 166.48)	159.50 (158.45 -160.54)	172.15 (171.21–173.09)
Weight, kg (mean, 95% CI)	79.39 (77.81 to 80.96)	77.57 (75.28–79.86)	81.23 (79.07–83.39)
Waist circumference, cm (mean, 95% CI)	96.47 (94.99 to 97.95)	97.49 (95.38–99.60)	95.44 (93.35–97.52)
BMI^4^, kg/m^2^ (mean, 95% CI)	28.76 (28.21 to 29.32)	30.30 (29.43–31.16)	27.22 (26.53–27.91)
BMI classification^5^ (%, 95% CI)			
Underweight	5.36 (3.81 to 7.49)	4.48 (2.60–7.59)	6.25 (4.01–9.60)
Normal	25.47 (22.02 to 29.25)	19.49 (15.37–24.39)	31.48 (26.12–37.40)
Overweight	27.80 (24.25 to 31.65)	25.84 (21.05–31.29)	29.76 (24.70–35.37)
Obese	41.38 (37.49 to 45.38)	50.19 (44.59–55.79)	32.51 (27.22–38.29)

^1^ The estimates for the variables *age group*, *sex*, *ethnic background* and *area* were derived prior to considering stratification (since these were the variables used to form the strata)

^2^ Other ethnicities contributed 2.00% (1.09 to 3.66%) to the Fijian of Indian descent or another category

^3^Hypertension classified as systolic blood pressure ≥ 140mmHg or diastolic blood pressure ≥ 90mmHg or self-report of taking medications for hypertension

^4^ BMI: Body mass index

^5^ BMI classification: *underweight* (< 18.5 kg/m^2^); *normal weight* (18.5 to 24.9 kg/m^2^); *overweight* (25.0 to 29.9 kg/m^2^); *obese* (≥ 30.0 kg/m^2^)

### Self-reported food insecurity in Fijian adults

**Fig. 1:**
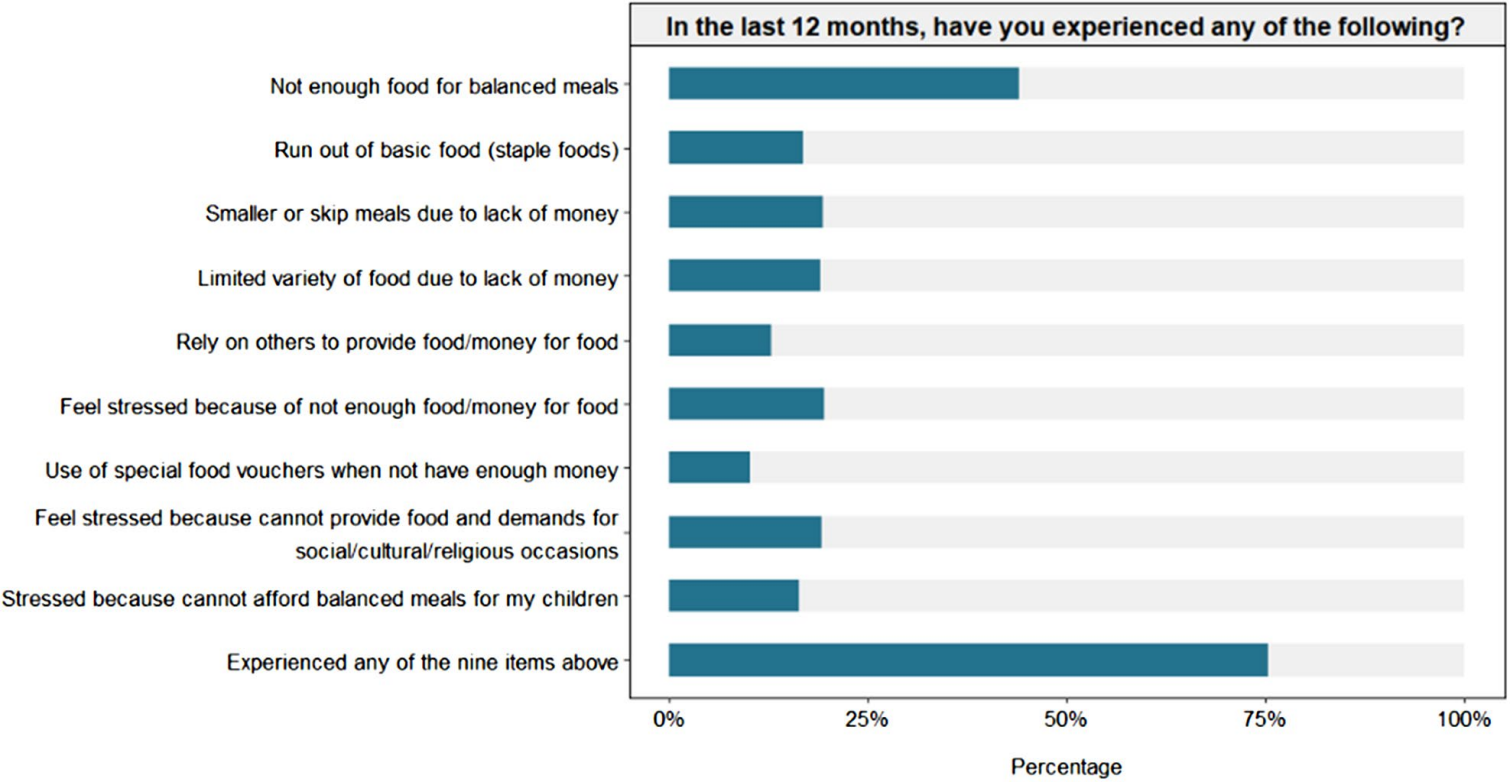
percentage of people reporting on different criteria for food insecurity

Three quarters of the people surveyed said they had experienced one or more of the nine indicators for food insecurity in the last 12 months (Table 2). 44% said that they had experienced not having enough food for balanced meals in the last 12 months. Nearly one fifth said that they had run out of staple foods (16.8%, 95% CI 13.9 to 20.2%), had to have smaller meals, or skip meals (19.3%, 16.2 to 22.9%) or limit variety of types of food due to lack of money (19.0%, 15.9 to 22.5%).A similar number of people said they felt stressed because of not enough money for food (19.5%, 16.4 to 23.0%), because they can’t meet demands for social/cultural/religious occasions (19.2%, 16.2 to 22.6%) or because they couldn’t afford balanced meals for their children (16.3%, 13.5 to 19.7%). 12.9% (10.4 to 15.9%) said they had to rely on others to provide money for food and 10.2% (7.9 to 13.0%) said they relied on special food vouchers (Fig. 1).

**Table 2:** Food security overall and by subgroups (weighted %, 95% CI)

	Overall	By sex		By age group		By area		By ethnicity	
		Female	Male	18 to 44 years	45 years and up	Deuba	Waidamudamu	ITaukei	FID and FOD
In the last 12 months, have you experienced any of the following?									
Not enough food for balanced meals^4^	44.0 (39.9 to 48.3)	42.2 (36.4 to 48.0)	45.9 (39.8 to 52.0)	43.6 (38.1 to 49.2)	44.7 (38.5 to 51.0)	41.0 (35.2 to 46.7)	48.6 (42.7 to 54.5)	38.4 (32.2 to 44.5)	48.9 (43.2 to 54.6)
Run out of basic food (staple foods)	16.8 (13.9 to 20.2)	17.0 (12.5 to 21.5)	16.6 (12.1 to 21.2)	17.1 (12.9 to 21.3)	16.3 (11.5 to 21.0)	14.9 (10.6 to 19.2)	19.6 (14.9 to 24.4)	18.6 (13.8 to 23.5)	15.2 (11.0 to 19.4)
Smaller or skip meals due to lack of money	19.3 (16.2 to 22.9)	17.7 (13.2 to 22.2)	21.0 (16.1 to 25.9)	21.0 (16.4 to 25.5)	16.4 (11.9 to 21.0)	17.7 (13.1 to 22.2)	21.8 (17.0 to 26.5)	17.3 (12.5 to 22.1)	21.1 (16.5 to 25.7)
Limited variety of food due to lack of money	19.0 (15.9 to 22.5)	19.1 (14.5 to 23.8)	18.9 (14.3 to 23.5)	20.6 (16.1 to 25.1)	16.2 (11.7 to 20.7)	17.0 (12.5 to 21.4)	22.0 (17.3 to 26.8)	18.9 (14.0 to 23.8)	19.1 (14.7 to 23.5)
Rely on others to provide food/money for food^1^	12.9 (10.4 to 15.9)	16.1 (11.6 to 20.5)	9.7 (6.4 to 12.9)	12.6 (9.0 to 16.1)	13.5 (9.1 to 17.8)	12.1 (8.5 to 15.8)	14.0 (9.8 to 18.2)	15.3 (10.8 to 19.9)	10.8 (7.5 to 14.1)
Feel stressed because of not enough food/money for food	19.5 (16.4 to 23.0)	20.0 (15.2 to 24.8)	19.0 (14.4 to 23.5)	21.8 (17.3 to 26.4)	15.4 (10.9 to 19.9)	18.2 (13.7 to 22.7)	21.4 (16.7 to 26.1)	22.6 (17.4 to 27.8)	16.8 (12.6 to 21.0)
Use of special food vouchers when not have enough money	10.2 (7.9 to 13.0)	12.2 (8.4 to 16.0)	8.1 (4.9 to 11.3)	8.3 (5.2 to 11.3)	13.5 (9.1 to 17.8)	8.9 (5.6 to 12.2)	12.1 (8.3 to 15.9)	9.2 (5.5 to 13.0)	11.0 (7.6 to 14.4)
Feel stressed because can’t provide food and demands for social/cultural/religious occasions	19.2 (16.2 to 22.6)	21.7 (16.8 to 26.7)	16.6 (12.5 to 20.7)	19.5 (15.3 to 23.7)	18.6 (13.7 to 23.5)	17.3 (13.1 to 21.6)	22.0 (17.1 to 26.9)	22.4 (17.2 to 27.6)	16.4 (12.4 to 20.4)
Stressed because can’t afford balanced meals for my children	16.3 (13.5 to 19.7)	17.3 (12.8 to 21.8)	15.4 (11.2 to 19.6)	17.0 (12.9 to 21.2)	15.1 (10.7 to 19.5)	15.7 (11.5 to 20.0)	17.2 (12.9 to 21.6)	17.8 (13.0 to 22.5)	15.1 (11.1 to 19.2)
Experienced any of the above	75.3 (71.4 to 78.8)	73.0 (67.7 to 78.3)	77.5 (72.3 to 82.7)	73.9 (69.0 to 78.9)	77.6 (72.3 to 82.9)	73.0 (67.7 to 78.2)	78.7 (73.9 to 83.5)	75.0 (69.5 to 80.6)	75.5 (70.5 to 80.5)
To what extent did the Covid-19 pandemic impact on your food security? **Greatly/somewhat (became more/slightly food insecure due to Covid-19)**^3,4^	66.9 (62.9 to 70.7)	70.4 (65.1 to 75.7)	63.4 (57.6 to 69.2)	66.7 (61.4 to 72.0)	67.3 (61.6 to 72.9)	62.7 (57.2 to 68.3)	73.2 (67.9 to 78.4)	55.2 (48.9 to 61.5)	77.0 (72.1 to 81.9)
Did you change what/how you ate or how you accessed food during the Covid-19 pandemic?									
Stocked up on food, more than you normally would have^4^	33.8 (30.0 to 37.9)	30.6 (25.2 to 36.0)	37.1 (31.3 to 42.9)	34.9 (29.7 to 40.2)	31.9 (26.0 to 37.7)	34.1 (28.6 to 39.6)	33.4 (27.9 to 38.9)	28.1 (22.4 to 33.8)	38.7 (33.2 to 44.2)
Made a home garden	67.8 (63.7 to 71.6)	69.7 (64.3 to 75.2)	65.9 (60.1 to 71.6)	67.1 (61.8 to 72.3)	69.1 (63.3 to 74.9)	69.7 (64.3 to 75.2)	64.9 (59.3 to 70.5)	69.8 (64.0 to 75.7)	66.0 (60.6 to 71.5)
Grew more fruits and vegetables to eat^3,4^	59.8 (55.6 to 63.8)	58.8 (53.1 to 64.5)	60.8 (54.9 to 66.8)	59.0 (53.5 to 64.5)	61.2 (55.1 to 67.3)	64.3 (58.6 to 70.0)	53.1 (47.2 to 58.9)	66.2 (60.1 to 72.2)	54.3 (48.6 to 59.9)
Changed what you ate – tried to eat more “health” foods	14.7 (12.0 to 18.0)	14.4 (10.1 to 18.6)	15.1 (10.9 to 19.3)	14.9 (11.0 to 18.8)	14.4 (9.9 to 19.0)	14.5 (10.5 to 18.6)	15.0 (10.7 to 19.3)	16.1 (11.4 to 20.8)	13.5 (9.7 to 17.4)
Ate less than you normally would have for health-related reasons	4.4 (3.0 to 6.3)	4.4 (2.0 to 6.7)	4.4 (2.1 to 6.6)	4.7 (2.5 to 6.9)	3.8 (1.4 to 6.1)	3.1 (1.1 to 5.0)	6.3 (3.5 to 9.2)	4.9 (2.3 to 7.6)	3.9 (1.9 to 5.9)
Ate less than you normally would have due to Covid-related food shortages^3^	9.9 (7.7 to 12.7)	9.7 (6.2 to 13.2)	10.1 (6.5 to 13.7)	10.4 (7.1 to 13.7)	9.0 (5.2 to 12.8)	6.9 (3.9 to 10.0)	14.4 (10.1 to 18.6)	9.7 (6.0 to 13.4)	10.1 (6.7 to 13.5)
Ate more than you normally would have	4.6 (3.1 to 6.8)	4.6 (2.1 to 7.2)	4.6 (2.0 to 7.1)	5.1 (2.6 to 7.6)	3.7 (1.4 to 6.0)	3.9 (1.6 to 6.3)	5.6 (2.9 to 8.4)	6.1 (3.0 to 9.1)	3.3 (1.3 to 5.4)

^1^ Significant difference by sex

^2^ Significant difference by age

^3^ Significant difference by area

^4^ Significant difference by ethnicity

### Perceived effect of COVID-19 on food insecurity

**Fig. 2:**
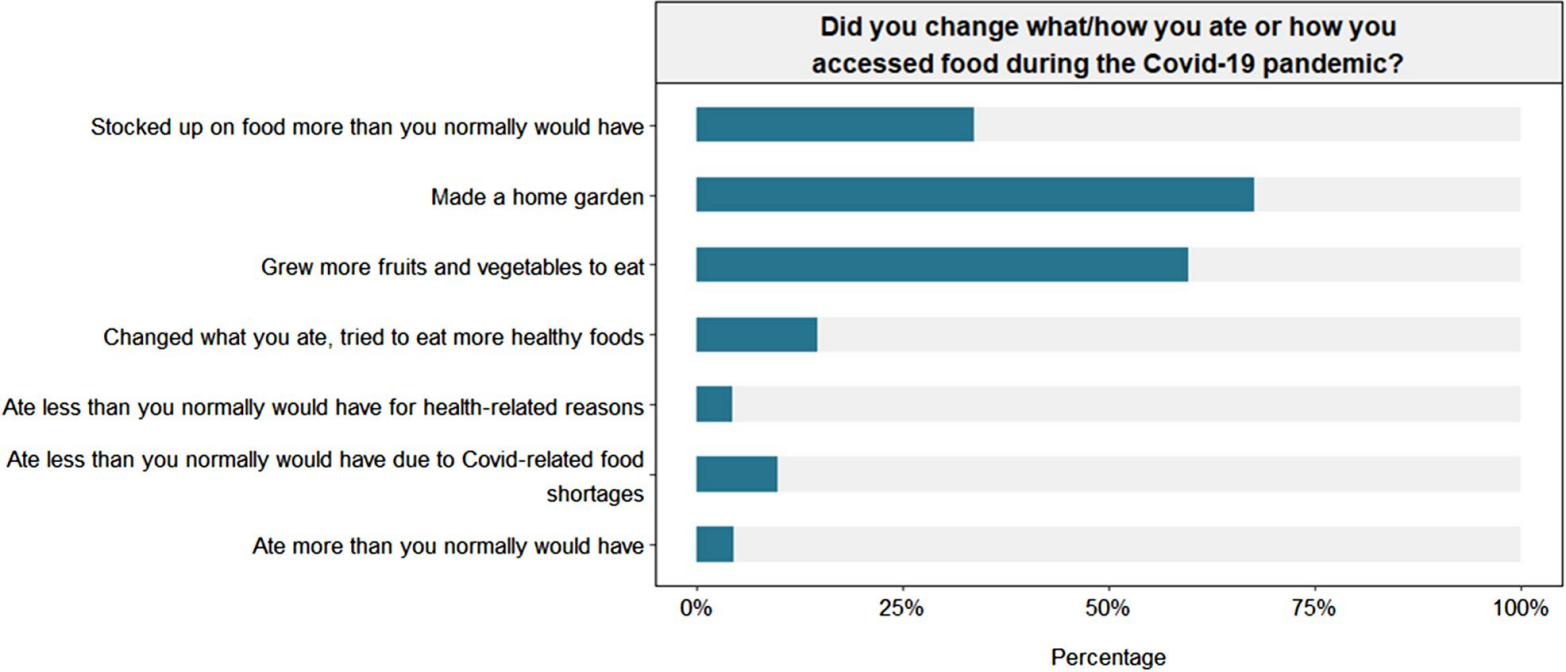
reported changes in food preparation and consumption due to COVID-19

70% of people said that they had become more food insecure due to the COVID-19 pandemic (Table 2). Respondents from Waidamudamu and respondents of Indian Fijian and other Fijian decent were more likely to report increasing food insecurity due to COVID-19. Many people reported changing what they ate, with most people saying they made a home garden (67.8%, 63.7 to 71.6%) or grew more fruits and vegetables (59.8%, 55.6 to 63.8%). Participants from Deuba and participants of iTaukei decent were more likely to report growing more fruit and vegetables than participants from Waidamudamu or those of Fijian Indian or other decent. Just over one third (33.8%, 30.0 to 37.9%) reported stocking up on food more than normal. Around 15% (14.7%, 12.0 to 18.0%) said that they tried to eat more “healthy” foods. Less than 5% (4.4%, 3.0 to 6.3%) said they ate less than they normally would for health reasons compared to 10% (7.7 to 12.7%) who reported eating less than they normally would because of COVID-19 related food shortages. Considerably more people from Waidamudamu (14.4%, 10.1 to 18.6%) reported eating less than normal due to COVID-19 related shortages compared to people from Deuba (6.9%, 3.9 to 10.0%). Less than 5% (4.6%, 3.1 to 6.8%) reported eating more than normal because of COVID-19 (Table 2; Fig. 2).

### Relationships between food insecurity and diet

There were no statistically significant associations between food insecurity or reported impact of COVID-19 on food insecurity and dietary intakes of salt, sugar and fruit and vegetables (See Table 3).

**Table 3:** **(i)** Relationship between food insecurity with salt intake

Salt intake	Unadjusted		Adjusted^1^	
	Mean (95% CI), g/day	*p*-value	Mean (95% CI), g/day	*p*-value
**Experienced food insecurity in the last 12 months**				
No	**5.85** (5.18 to 6.51)	0.263	**5.93** (5.26 to 6.60)	0.171
Yes	**5.42** (5.10 to 5.75)		**5.41** (5.09 to 5.73)	
**Experienced a change in food security due to Covid-19**				
Did not experience a change in food security	**5.29** (4.81 to 5.77)	0.258	**5.17** (4.70 to 5.64)	0.084
Became more or slightly food insecure	**5.64** (5.27 to 6.01)		**5.72** (5.34 to 6.11)	

^1^ Adjusted for sex, age, enumeration area, ethnicity, highest level of education, BMI classification, and hypertension status

**Table 3:** **(ii)** Relationship food insecurity with sugar intake

Sugar intake	Unadjusted		Adjusted^1^	
	Mean (95% CI), g/day	*p*-value	Mean (95% CI), g/day	*p*-value
**Experienced food insecurity in the last 12 months**				
No	**78.86** (67.38 to 90.34)	0.604	**79.70** (68.14 to 91.26)	0.685
Yes	**82.68** (74.22 to 91.15)		**82.75** (74.20 to 91.31)	
**Experienced a change in food security due to Covid-19**				
Did not experience a change in food security	**81.87** (69.02 to 94.72)	0.983	**75.04** (62.49 to 87.60)	0.203
Became more or slightly food insecure	**81.69** (73.40 to 89.99)		**85.54** (76.60 to 94.47)	

^1^ Adjusted for sex, age, enumeration area, ethnicity, highest level of education, BMI classification, and hypertension status

**Table 3:** **(ii)** Relationship food insecurity with sugar intake

Sugar intake	Unadjusted		Adjusted^1^	
	Mean (95% CI), g/day	*p*-value	Mean (95% CI), g/day	*p*-value
**Experienced food insecurity in the last 12 months**				
No	**78.86** (67.38 to 90.34)	0.604	**79.70** (68.14 to 91.26)	0.685
Yes	**82.68** (74.22 to 91.15)		**82.75** (74.20 to 91.31)	
**Experienced a change in food security due to Covid-19**				
Did not experience a change in food security	**81.87** (69.02 to 94.72)	0.983	**75.04** (62.49 to 87.60)	0.203
Became more or slightly food insecure	**81.69** (73.40 to 89.99)		**85.54** (76.60 to 94.47)	

^1^ Adjusted for sex, age, enumeration area, ethnicity, highest level of education, BMI classification, and hypertension status

## Discussion

This study reported on a food security survey conducted in 2022. The results show high levels of food insecurity in the central division of Fiji, with people perceiving that their food security was effected by the COVID-19 pandemic. Further, there was no observed cross-sectional association between food insecurity and salt, sugar, fruit or vegetable intake. This study contributes to the public health nutrition and food security literature by updating evidence on food security in Fiji and providing new information on the perceived effect of COVID-19. This is important information for evidenced based policy making in Fiji, and of relevance to the Pacific Region more broadly as they strive to meet the UN Sustainable Development Goals of “Zero Hunger” and “Good Health and Wellbeing” [27].

This study contributes to the literature that reports on the global issue of food insecurity, exaggerated by COVID-19 [28]. Throughout the COVID-19 pandemic, countries reported disruptions to food supply chains that impacted food availability and accessibility and economic pressures that have effected consumer purchases [29]. Our findings are similar to those reported in studies from India [30], People’s Republic of China [31], and Papua New Guinea [29] describing unprecedented levels of food insecurity related to the COVID-19 pandemic. The evidence generated by this study is an important step to understanding the impact of COVID-19 on food security in Pacific Island Countries. These data are relevant to filling an evidence gap particularly in view of policy development for resilient food systems that may be influenced by future challenges to food security.

### Covid-19 increased food insecurity but led to some positive dietary behaviors

Our study showed that 70% of people experienced increased food insecurity related to the COVID-19 pandemic. However, several food security enhancing behaviors were reported including growing more fruit and vegetables and trying to eat more healthily. This is in line with another study on the impact of COVID-19 in Fiji, which demonstrated a shift to subsistence farming [3]. Studies from other countries have reported positive changes in dietary practices related to the COVID-19 pandemic. For example, a study in Peru demonstrated an increase in breastfeeding amongst mothers and a reduction in sweet food consumption by infants and young children [32]. An anthropologist assessing the impact of the COVID-19 pandemic on food insecurity in Fiji, attributed positive changes, such as those observed in our study, to the “Vanua” concept of togetherness [33]. Vanua means tribe or clan but implies a sense of duty to look after each other, which during times of scarcity means sharing food. At a government level, the Ministry of Agriculture’s home gardening scheme supported this concept of Vanua by distributing almost 12,000 seed packages across Fiji in 2020 [33] with the goal of increasing self-sufficiency. Several studies from other countries have demonstrated that home gardening schemes can improve fruit and vegetable consumption [3, 34, 35].We recommend that future initiatives build on this momentum as part of a coordinated approach across the food supply chain to reverse the decline in fruit and vegetable consumption and concurrently improve food security in Fiji [36].

### Ultra-processed foods are displacing traditional foods and increasing NCDs

In line with other studies [11, 37, 38], our study showed high levels of self-reported food insecurity and poor nutrient intakes (high salt and sugar and low fruit and vegetable intake). This is in keeping with a Pacific wide transition from traditional foods including local fruits and vegetables towards cheaper, highly processed foods, high in salt and sugar [37]. Collectively, these studies suggest that ultra-processed foods are reducing diet quality and contributing to increasing non-communicable diseases [39]. In addition to boosting food security and self-sufficiency, the Ministry of Agriculture’s home gardening scheme may also improve health if home grown foods replace ultra-processed foods in the population’s diet.

### Lack of association between food security and dietary intakes

The absence of an association between food security and dietary intake of salt and sugar or fruit and vegetables in our study is likely because most people were eating diets high in salt and sugar and low in fruit and vegetables more generally [17]. The lack of association may also be due to poor sensitivity of the dietary assessment tool to examine associations between diet and food insecurity. Nutrient intakes were measured based on a single 24-hour dietary survey. Single 24-hour diet recalls have limitations in their ability to assess habitual diet intake accurately, meaning they are not the best approach for looking at associations between dietary intake and other variables at an individual level [40, 41]. However, a single 24-hr diet recall was used in this case for the purpose of assessing population level intake, and to provide a measure for future monitoring. Other studies have shown a clear relationship between food insecurity and diet quality. A study of low-income adults in the US, which used a similar self-reported method of measuring food insecurity but looked at diet quality rather than nutrient intake, showed an association between food insecurity and diet quality, but no association with overall energy intake in low-income adults [42]. The UN report on the State of Food Security and Nutrition in the World, identified studies in Tanzania and Ghana showing that people who were food insecure (based on their answers on the Food Insecurity Experience Scale, a similar tool to the one used in the present study) reported consuming less diverse diets and fewer nutritious foods that contribute to healthy diets [43]. These studies used questionnaires which focused on diet quality and variety in line with food security, rather than comparing measures of food insecurity with reported absolute intakes of nutrients or overall amounts of fruits and vegetables, which may be a reason why they identified an association between food insecurity and diet, where we did not. It is also possible that using other diet assessment methods such as food frequency questionnaires, to gain a better estimate of habitual intake compared to our use of 24-hour diet recall, may have yielded different results. There is a need for ongoing monitoring of diet quality and food security in Fiji at a national level. In the future, the relationship between dietary intake and food security may be more sensitively assessed using the Diet Quality Questionnaire (DQ-Q), developed by the Global Diet Quality Project [44] to explore associations between food insecurity and diet. While food insecurity was measured based on experiences over the last 12 months, it was self-reported, and we used a composite variable that may have obscured associations with aspects of food security. We also categorized participants into two groups and may have missed subtle differences in food security between participants.

### Impact of climate change on food security

This study did not investigate climate change specifically, however, the World Health Organization has identified food security as a high priority climate-sensitive health risks in the Pacific Islands [45]. It is therefore likely that the changing climate has had an impact on the presented results. This is in line with previous qualitative work in Fiji which found that people perceive climate change over the past generations has led to unhealthy eating practices [46]. In the Pacific region there is a dependency on agricultural practices to produce affordable and easily accessible foods [47]. Climate change issues such as rising water levels, cyclones, storm surges, heat stress and drought can undermine food production and effective supply of nutritious foods [47]. Poor water quality is another climate-sensitive factor that could have impacted our findings, as poor water quality both reduces the potential for sustainable food production and can also impact on people’s ability to purchase (if they are spending money on bottled water) or prepare (if there is no water available for cooking) nutritious food, further forcing the move towards processed packaged foods [48]. Therefore, we propose that future work focuses on monitoring food security in parallel to work to protect the climate and support water security in the Pacific.

### Strengths and weaknesses

Key strengths of this study are the large sample drawn from two areas in the Central Division of Fiji, the fact that comprehensive up-to-date household listing was undertaken as a basis for sampling and the high response rate, meaning that the sample was very likely to be representative of the Central Division of Fiji. The study is also part of a broader program of work on strengthening and monitoring food policy interventions in Fiji. This includes representatives from Government Ministries (Ministry of Health) and technical support agencies including the World Health Organization and the Food and Agriculture Organization of the United Nations. As such, findings have been made available to relevant policy makers and Fiji’s development partners [49].

A key weakness is that the survey did not cover the outer islands where levels of food insecurity are thought to be higher. While we acknowledge that climate change and water security may be important factors in interpreting our findings, we did not collect information on these factors, and suggest that this is an important focus for future studies. We assessed diet based on a single 24-hr diet recall and only assessed relationships between food insecurity and key nutrients in line with the overarching program of work that focuses on reducing diabetes and hypertension [16]. Other diet assessment methods, for example food diaries, may have captured usual diet better although are more burdensome for participants [50]. As with any self-reported measure, there is likely to be an element of social desirability bias, which may mean that participants were less likely to report unhealthy foods consumed and may have under-reported measures for food insecurity. Similarly, we cannot rule out recall bias, where participants may not have clearly recollected their dietary intake, albeit we aimed to reduce the risk of this bias by following the multiple pass diet recall methodology. It is possible that the lack of association observed between food security and the diet measures could have been due to confounding bias, given the cross-sectional and observational nature of this study. Complete case analyses were conducted; however, the level of missing data is unlikely to have created a bias in the estimates, albeit our approach to missing data assumed that missingness from removing implausible values was completely at random. In terms of statistical analyses used, linear regression models were used to explore the relationship between food insecurity and the markers of dietary intake, and chi-square tests to compare proportions. A limitation of the linear regression analysis is the pooling of the nine food security questions into a single measure. Nonetheless, looking at the relationship of each of the food security question with intakes was not conducted, to avoid doing multiple significance testing and inflation of type I error. Adjustments for multiple testing could have been conducted for the chi-square test however this was not a component of the study protocol and as such is a limitation. The food insecurity questions were adapted from the 2015 Fijian National Food and Nutrition Survey with a view to being able to assess changes over time in Fiji, rather than being based on tools or questionnaires used in other countries. It is therefore not possible to compare levels of food insecurity in Fiji with other countries based on this assessment. Also, we did not test the reliability of these adapted questions. Future efforts to tackle food insecurity need to simultaneously address diet quality including increasing fruit and vegetable consumption and decreasing salt and sugar intake. Fiji’s Food and Nutrition Security Action Plan, which is yet to be endorsed by government in Fiji, includes complementary policies to tackle both issues.

## Conclusion

Based on participants self-reported experiences, food insecurity was high and was influenced by COVID-19, but no associations were observed between self-reported food insecurity and dietary intakes. Some of the adaptive responses by individuals such as growing more fruit and vegetables should be fostered by government through initiatives such as strengthening existing program around the provision of seeds. More detailed studies to obtain nationally representative data on food security and quality are urgently needed to inform future food policy initiatives.

### Electronic supplementary material

Below is the link to the electronic supplementary material.

**Supplementary Material 1:** Alignment of study reporting with “The Strengthening the Reporting of Observational Studies in Epidemiology (STROBE) statement: guidelines for reporting observational studies”

## Data Availability

The datasets used and/or analysed during the current study are available from the corresponding author on reasonable request.
