# Supplementary material for: Food insecurity, COVID-19 and diets in Fiji – a cross-sectional survey of over 500 adults
Source: Global Health. 2023 Dec 11;19:99. doi: 10.1186/s12992-023-01004-w (PMC10712026; doi:10.1186/s12992-023-01004-w)
Supplement: Supplementary file 1 — Supplementary Material 1: Alignment of study reporting with “The Strengthening the Reporting of Observational Studies in Epidemiology (STROBE) statement: guidelines for reporting observational studies” [file 12992_2023_1004_MOESM1_ESM.docx]

**Supplementary material.** Webster et al. Food insecurity, COVID-19 and diets in Fiji – a cross-sectional survey of over 500 adults**.**

**Supplementary Table 1.** Alignment of study reporting with “The Strengthening the Reporting of Observational Studies in Epidemiology (STROBE) statement: guidelines for reporting observational studies” (1, 2)

|  | Item No | Recommendation | Page No |
| --- | --- | --- | --- |
| **Title and abstract** | 1 | (*a*) Indicate the study’s design with a commonly used term in the title or the abstract: “**Food insecurity, COVID-19 and diets in Fiji – a cross-sectional survey of over 500 adults”** | 1 |
|  |  | (*b*) Provide in the abstract an informative and balanced summary of what was done and what was found: **Abstract provided** | 2 |
| Introduction | | | |
| Background/rationale | 2 | Explain the scientific background and rationale for the investigation being reported: **See introduction section** | 4-5 |
| Objectives | 3 | State specific objectives, including any prespecified hypotheses: **“The primary aim of this paper was to describe self-reported food security in Fijian adults aged 18 years or older, including how consumption patterns may have changed in response to COVID-19. A secondary aim was to examine associations between food security and dietary intakes.”** | 5 |
| Methods | | | |
| Study design | 4 | Present key elements of study design early in the paper: **“This was a cross-sectional study within the central division of Fiji”** | 5 |
| Setting | 5 | Describe the setting, locations, and relevant dates, including periods of recruitment, exposure, follow-up, and data collection: **Covered in the “Sample size and recruitment” section** | 5 |
| Participants | 6 | (*a*) Give the eligibility criteria, and the sources and methods of selection of participants**: Eligibility criteria base on age (18 years or older), and ability to provide informed consent. Further information contained in “Sample size and recruitment” section.** | 5-6 |
| Variables | 7 | Clearly define all outcomes, exposures, predictors, potential confounders, and effect modifiers. Give diagnostic criteria, if applicable. **Defined in “Data analyses” section.** | 7 |
| Data sources/ measurement | 8* | For each variable of interest, give sources of data and details of methods of assessment (measurement). Describe comparability of assessment methods if there is more than one group. **Described in “Survey Measures” section.** | 6-7 |
| Bias | 9 | Describe any efforts to address potential sources of bias. **Described throughout methods section, including in steps taken to identify participants (randomisation), the collection of survey data, and analyses conducted.** | 5-8 |
| Study size | 10 | Explain how the study size was arrived at**. Described in “Sample size and recruitment” section.** | 5 |
| Quantitative variables | 11 | Explain how quantitative variables were handled in the analyses. If applicable, describe which groupings were chosen and why. **Descried in “Data-analysis” section.** | 7 |
| Statistical methods | 12 | (*a*) Describe all statistical methods, including those used to control for confounding | 7 |
|  |  | (*b*) Describe any methods used to examine subgroups and interactions | 7 |
|  |  | (*c*) Explain how missing data were addressed. **Complete case analysis conducted, 45 people had missing or implausible data and were excluded from analysis.** | 7 |
|  |  | (*d*) If applicable, describe analytical methods taking account of sampling strategy. | 7 |
|  |  | (*e*) Describe any sensitivity analyses | 7 |
| Results | | | |
| Participants | 13* | (a) Report numbers of individuals at each stage of study—eg numbers potentially eligible, examined for eligibility, confirmed eligible, included in the study, completing follow-up, and analysed. **534 people participated.** | 7 |
|  |  | (b) Give reasons for non-participation at each stage. **Reasons for non-response or refusal included: participant had permanently moved (n=50), refused to participate (no specific reason given, n=46), to busy to participate (n=15), family objected to participation (n=4), deceased (n=3) or unwell (n=3).** | 7-8 |
|  |  | (c) Consider use of a flow diagram. **Only one stage for data collection, flow diagram not provided.** | NA |
| Descriptive data | 14* | (a) Give characteristics of study participants (eg demographic, clinical, social) and information on exposures and potential confounders. **Provided in the section on “demographics and health status” and in Table 1.** | 7 |
|  |  | (b) Indicate number of participants with missing data for each variable of interest. **Complete case analysis conducted, 45 people had missing or implausible data and were excluded from analysis.** | 7-8 |
| Outcome data | 15* | Report numbers of outcome events or summary measures. **Table 2.** | Table 2 |
| Main results | 16 | (*a*) Give unadjusted estimates and, if applicable, confounder-adjusted estimates and their precision (eg, 95% confidence interval). Make clear which confounders were adjusted for and why they were included | Table 3. |
|  |  | (*b*) Report category boundaries when continuous variables were categorized | NA |
|  |  | (*c*) If relevant, consider translating estimates of relative risk into absolute risk for a meaningful time period | NA |
| Other analyses | 17 | Report other analyses done—eg analyses of subgroups and interactions, and sensitivity analyses: **Reported results section** | 7-8 |
| Discussion | | | |
| Key results | 18 | Summarise key results with reference to study objectives. **Opening paragraph of the discussion.** | 9 |
| Limitations | 19 | Discuss limitations of the study, taking into account sources of potential bias or imprecision. Discuss both direction and magnitude of any potential bias. **Discussed in the strengths and limitations section.** | 11 |
| Interpretation | 20 | Give a cautious overall interpretation of results considering objectives, limitations, multiplicity of analyses, results from similar studies, and other relevant evidence. **Discussion section.** | 9-12 |
| Generalisability | 21 | Discuss the generalisability (external validity) of the study results. **Discussed in strengths and limitations section**. | 11 |
| Other information | | | |
| Funding | 22 | Give the source of funding and the role of the funders for the present study and, if applicable, for the original study on which the present article is based. **Stated in “Funding” section of the Declarations section** | 12-13 |

**References:**

1. Von Elm E, Altman DG, Egger M, Pocock SJ, Gøtzsche PC, Vandenbroucke JP. The Strengthening the Reporting of Observational Studies in Epidemiology (STROBE) statement: guidelines for reporting observational studies. The Lancet. 2007;370(9596):1453-7.

2. Equator Network. The Strengthening the Reporting of Observational Studies in Epidemiology (STROBE)Statement: guidelines for reporting observational studies 2023 [Available from: <https://www.equator-network.org/reporting-guidelines/strobe/>.
